# Health needs, access to healthcare, and perceptions of ageing in an urbanizing community in India: a qualitative study

**DOI:** 10.1186/s12877-017-0544-y

**Published:** 2017-07-19

**Authors:** Nandita Bhan, Pavitra Madhira, Arundati Muralidharan, Bharati Kulkarni, GVS Murthy, Sanjay Basu, Sanjay Kinra

**Affiliations:** 1 Public Health Foundation of India, Plot 47, Sector 44, Gurgaon, India; 2 Indian Institute of Public Health Hyderabad, Hyderabad, India; 3 WaterAid in India, Delhi, India; 4 National Institute of Nutrition, Hyderabad, India; 5 Department of Clinical Research, London School of Hygiene & Tropical Medicine, London, UK; 6 Stanford Prevention Research Center, Palo Alto, USA; 7 Clinical Epidemiology, London School of Hygiene & Tropical Medicine, London, UK

**Keywords:** Ageing, Elderly, India, Geriatric care

## Abstract

**Background:**

India’s elderly population is rising at an unprecedented rate, with a majority living in rural areas. Health challenges associated with ageing, changing social networks and limited public health infrastructure are issues faced by the elderly and caregivers. We examined perceptions of health needs of the elderly across local stakeholders in an urbanizing rural area.

**Methods:**

The qualitative study was conducted among participants in the Andhra Pradesh Children and Parents Study (APCAPS) site in Rangareddy district, Telangana. We collected data using focus group discussions and interviews among communities (*n* = 6), health providers (*n* = 9) and administrators (*n* = 6). We assessed stakeholders’ views on the influence of urbanization on health issues faced and interventions for alleviating these challenges. We used a conceptual-analytical model to derive themes and used an inductive approach to organizing emerging codes as per a priori themes. These were organized as per thematic groups and ranked by different authors in order of importance. Bronfebrenner’s theory was used to understand stakeholder perspectives and suggest interventions within four identified spheres of influence - individual, household, community and services.

**Results:**

Stakeholders reported frailty, lack of transport and dependence on others as factors impacting health access of the elderly. Existing public health systems were perceived as overburdened and insensitive towards the elderly. Urbanization was viewed positively, but road accidents, crime and loneliness were significant concerns. Interventions suggested by stakeholders included health service outreach, lifestyle counseling, community monitoring of healthcare and engagement activities.

**Conclusions:**

We recommend integrating outreach services and lifestyle counseling within programs for care of the elderly. Community institutions can play an important role in the delivery and monitoring of health and social services for the elderly.

**Electronic supplementary material:**

The online version of this article (doi:10.1186/s12877-017-0544-y) contains supplementary material, which is available to authorized users.

## Background

The population of the elderly (60 years or more) is rising at an unprecedented rate in India [1, 2]. In 2013, the elderly comprised 8% of India’s population and this is projected to rise to 18.3% in 2050 [1–3]. Globally, ageing is associated with multiple morbidities, particularly the rise of cardiovascular diseases, physical impairments and mental health conditions [4–7]. Hence, the increasing population of the elderly in India will lead to greater morbidity in the future [8, 9]. Data show that the age composition of rural areas is changing with an increase in the proportion of the elderly residing in rural areas [10]. This residential pattern is attributed to the out-migration of young persons to urban areas and to the in-migration of the elderly after retirement to rural areas [10, 11]. It will also likely lead to changes in the economic, social and cultural life of the community. Further, these rural settings are often limited in health services and other infrastructure, specifically geared towards care of the elderly [12]. This adds to challenges of healthcare for the elderly as health systems are not prepared or empathetic to the needs of this population group.

Over the past decade, socioeconomic transitions such as urbanization are changing physical infrastructure and social relations in rural areas in India [13, 14]. Urbanization is transforming rural economies, socioeconomic status and transport systems, which may influence morbidity and access to health services. Greater incomes and transition to non-farm economies will impact the types of foods consumed and levels of physical activity, thereby increasing chronic disease risks. At the same time, changing family structures^1^ (from joint families or households with multiple generations cohabiting to nuclear or single generation families), community norms and traditional social networks are influencing the resources and care for the elderly in settings undergoing urban transition [11, 15, 16]. The influence of these changes on the health needs of the elderly and experiences of health challenges faced by the elderly are not well understood.

India introduced the National Policy on Older Persons to highlight priority domains for wellbeing of the elderly in 1999 [12]. Subsequently in 2011, a National Program for Health Care of the elderly was formulated which aims at developing geriatric care in India [17]. These policies are at early roll-out stages which offer a unique window of opportunity to strengthen their design and implementation by incorporating perspectives and experiences of local implementers and beneficiaries of these services. Incorporating the perspectives of these local stakeholders can aid in midcourse corrections and in preventing policies from being misdirected.

In this study, our aim was to understand perceptions of health needs of the elderly across three key local stakeholder groups (community, health providers and administrators) in an urbanizing rural area. We also assessed stakeholders’ view of the influence of urbanization on health needs and challenges faced by the elderly. Finally, our objective was also to identify a few key interventions or health services that these stakeholders believed would meet these health needs of the elderly.

## Methods

In line with these objectives, we conducted a qualitative study in the villages of Rangareddy district in Telangana, which is the field site of the Andhra Pradesh Children and Parents Study (APCAPS) [18]. APCAPS is a birth cohort of households in twenty nine villages that participated in a community nutrition trial in 1987–90. Follow-ups of the households have investigated the development of chronic diseases in the community and associated anthropometric, cardio-metabolic and socioeconomic factors [19–22]. Rangareddy district is located in the southern Indian state of Telangana and is urbanizing rapidly due to its proximity to Hyderabad city. The degree of urbanization in APCAPS villages is captured by a metric of ‘*urbanicity*’ which is based on field surveys and geospatial data [23]. This urbanicity is based on night time light data from the NOAA Defence Meterological Satellite Programme (DMSP) using observations from space. These lights include outside lights linked with human settlements and were estimated using Stable Light product which consists of yearly average light intensity measures processed and filtered to reduce contamination. Urbanicity as a metric represents a proxy for human settlements, energy use and economic activity. A night time light intensity urbanicity score was estimated for each village and standardized by village size and areas using GPS-based ground surveying. Values for NTLI were summed over the area of each village and classified into low, medium and high urbanicity tertiles. The metric has been validated [23].

**Fig. 1:**
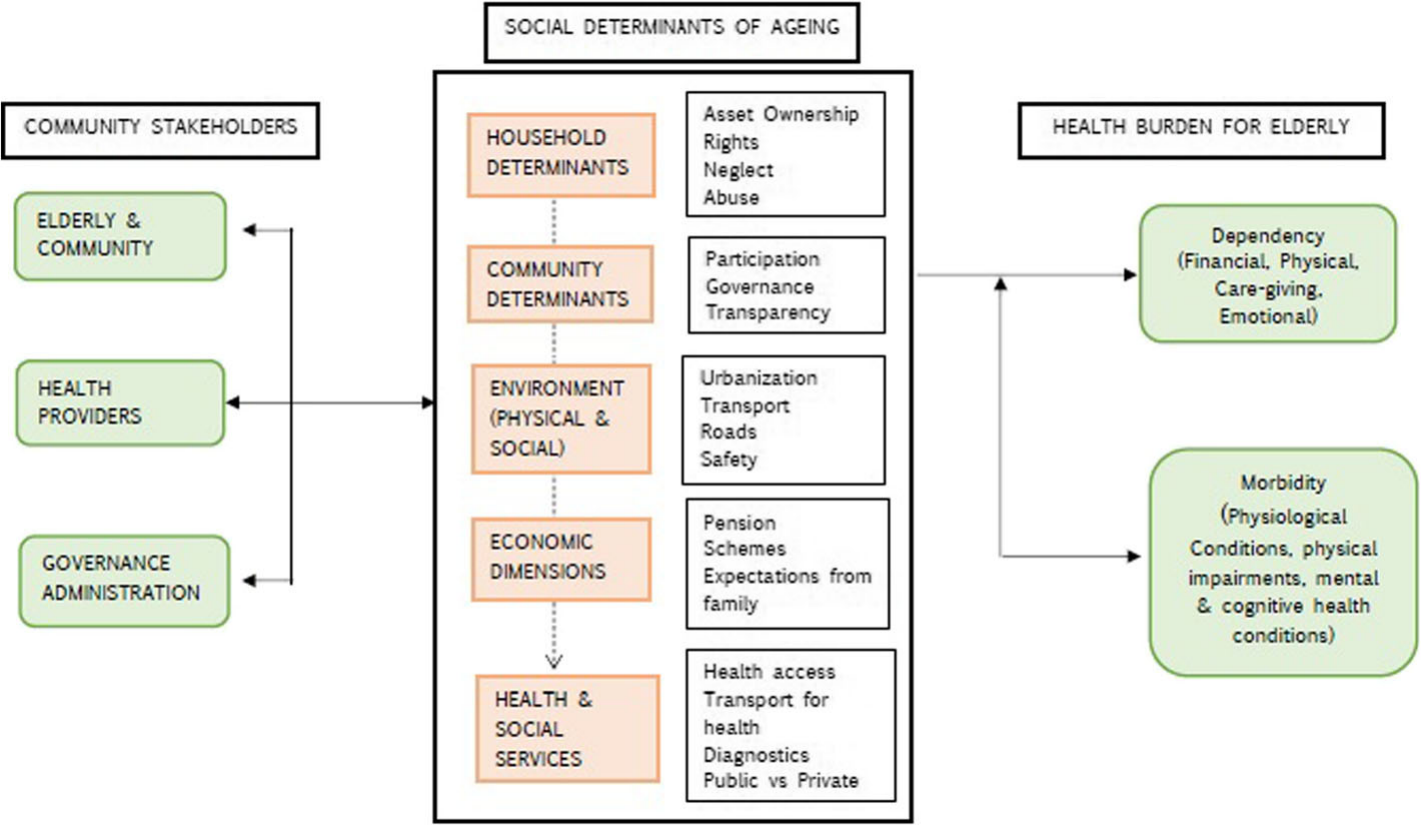
Social Determinants of Ageing: Conceptual-analytical model linking multiple determinants to morbidity and dependency for the elderly in APCAPS villages, Rangareddy district, India

For the study, we developed a conceptual-analytical framework based on existing models on social determinants of health [24, 25] (Fig. 1). This conceptual framework defined five themes of focus that were of relevance to health of the elderly. These included household issues, community factors, physical and social environments, economic aspects and health and social services. These were used for instrument development for each stakeholder group. This conceptual framework was also used to guide the analyses and presentation of findings. Several sub-themes and codes emerged from the data analysis that were related to these five broad thematic spheres. Some of these are presented in Fig. 1.

In this study, we focused data collection on three stakeholder groups (details in Table 1). The first group comprised *community members*. We conducted six focus group discussions (FGDs) among the community (each focus group comprising 7–10 members)) to elicit perceptions of health needs of the elderly and related determinants. The use of focus group discussions helped in assimilating and comparing perspectives across diverse groups in the villages, including the elderly, family members and other villagers.

**Table 1:** Sample details and research focus of the qualitative study on health needs among the elderly in the APCAPS villages, Rangareddy district, India

Participant type	Sampling details (Levels of Urbanicity)	Age and gender profile	Focus of FGDs/interviews
Community (the elderly, family members and other villagers)	*N* = 6 (Low =3, Moderate =1, High =2) Group size (7–10 members)	Age range: 40–80 years. Equal participation by men and women; in one FGD more women compared to men.	Health needs of the elderly, economic and social dependence on families, changing physical and social environment and access of health services.
Health Providers (government doctor, registered medical practitioner, auxiliary nurse midwife, pharmacist and 108 service^a^)	*N* = 9 (Low =2, Moderate =3, High =4) Individual interview (except in one case of interview of midwife, pharmacist and 108 service provider)	Age range: 25–52 years. Only 2 providers were female.	Health needs of the elderly, health and social services for the elderly in the villages and barriers (physical, financial and social).
Administrators	*N* = 6 (Low =4, Moderate =1, High =1) Individual interview	Age range: 30–40 years. Only 1 administrator was female	Health and social service programs, changing physical and social environments in the villages, engagement of the elderly in community activities.

^a^108 is an ambulatory service provided by a public private partnership similar to the 104 preventive health van. Villagers dial 108 from their mobile phones in case of ambulatory assistance needed

The second group comprised public and private *health providers* in the villages. These providers included primary health care medical professionals, private registered medical practitioners and ancillary health providers. We conducted semi -structured interviews (*n* = 9) to understand health systems challenges and the perceptions of providers on health needs of the elderly. The use of semi-structured interviews allowed the use of probes to draw out responses from health providers that flowed naturally and addressed key study themes.

The third group comprised local governance *administrators* and included village leaders and panchayat (village council) representatives. We conducted brief structured interviews (*n* = 6) to assess administrator perspectives on health needs of the elderly in the community, engagement of the elderly in community activities and challenges with delivery of health and social services. These interviews allowed the triangulation of themes from focus groups and for considering feasible potential interventions focused at the elderly in the community.

Data collection instruments (topic guides) for FGDs and interviews were based on study objectives, previous research and the conceptual framework. In addition to structured questions, probes were added to get further information on key aspects. Areas of inquiry for the topic guide included profile of the community, health related services and issues, free-listing of health issues of importance, issues related to access of health services (e.g. care, diagnostics, medicines), access, quality and interactions with private versus public health services, dependency (physical and financial) on others and the influence of urbanization on community and the elderly. Data were collected by two teams of two field investigators who received training in qualitative data collection. FGDs were conducted in offices of *gram sabhas* 2 and interviews were conducted in clinics, homes or offices of respondents as per their convenience.

Data collection was stratified by urbanicity of the study villages to elicit a range of perspectives. Following sampling of villages for FGDs, community members were invited at random to participate participating in discussions. Details on sampled villages stratified by urbanicity is available in Additional file 1: Table S1. From the 29 villages in the APCAPS study site, data were collected from four low urbanicity, two medium urbanicity and 6 high urbanicity villages. These villages were identified randomly to represent a range of two key characteristics – population (as per a 2013 survey) and distance from the city (Hyderabad). Interviewees were chosen to conrepresent a mix of participant occupations, sector (public or private), gender and level in the administrative hierarchy.

Participants in the FGDs were older (age range from 40 to 85 years) with comparable gender distribution except in one group, which comprised more women. In comparison, participants in the health and governance systems interviews were younger (25–52 years) with more men compared to women. FGDs and interviews were conducted in the local language (Telugu). These were translated and transcribed by field staff. On average, FGDs lasted one hour fifteen minutes and interviews lasted about thirty minutes. We obtained informed consentto conduct and audiotape FGDs and interviews. Confidentiality and privacy of the participants were ensured at all times. Data on participant identifiers were only available to the core study team; we did not report village names, and analysed and reported by village urbanicity. The study received ethical approval by research ethics committees at the Public Health Foundation of India, the National Institute of Nutrition (India) and the London School of Hygiene and Tropical Medicine.

**Fig. 2:**
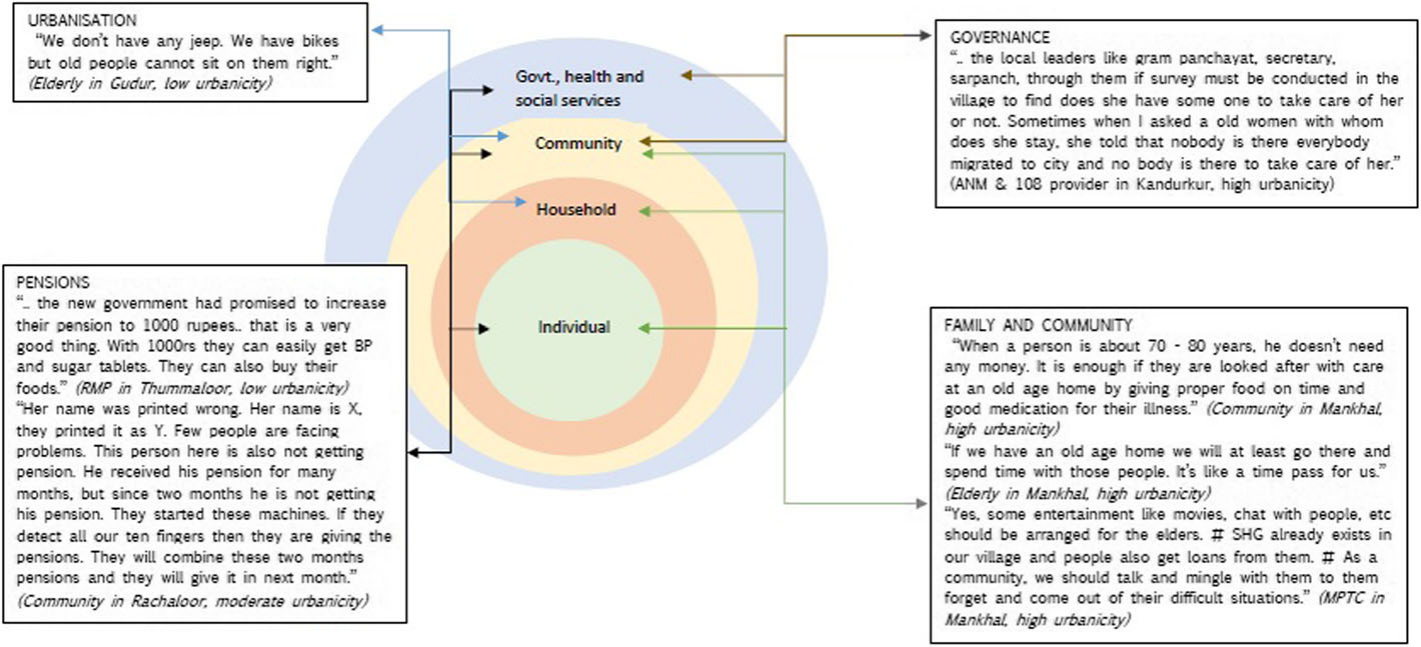
Interventions suggested by stakeholders to improve health of the elderly classified as per Bronfenbrenner’s Ecological Systems approach in APCAPS villages, Rangareddy district, India

We analyzed data using the conceptual-analytical model (Fig. 1) and Urie Bronfenbrenner’s Ecological Systems Theory [26]. Bronfenbrenner’s Ecological Systems Theory assesses the influence of context on an individual’s development. The theory, originally developed to understand child development, provides a broader view of human development. It frames an individual as being nested within multiple spheres of influence (systems) – microsystems, mesosystems, exosystems and macrosystems. The model provides a complex view of the individual and individual behavior, which is considered bounded by circumstances and the interaction of different systems. The theory has mostly been applied by those working on child development, and applications in the field of ageing are few. Contrary to interpretations from the field of development psychology, in understanding ageing, greater agency can be attributed to an older person – who is not only influenced by circumstances, but is actively engaged in shaping their environment, responding to them and evoking responses from different stakeholders [27]. In this study, there was an added challenge of dynamism introduced by the changing context of urbanization which will influence health and development of the elderly person. Analytical themes were derived from the conceptual analytical model. Two authors independently hand-coded the FGD and interview manuscripts using Microsoft Excel and emerging codes were organized as per a priori themes from the conceptual framework (Fig. 1). The codes emerging from data were related to these five broad themes. These were reviewed by the third author for agreement and in case of disagreement, further discussion among the authors guided their allocation to a thematic group. Codes that were agreed on were placed within each thematic group (Fig. 1). Identified quotes from the transcripts were ranked by the authors in the order of importance and where ideas overlapped, authors retained only one quote. Bronfenbrenner’s theory was used to understand stakeholder perspectives on what can change in the community and categorize suggested interventions within spheres of influence. It was also used to define the different systems (spheres), and quotes relating to potential interventions were matched with the four identified spheres - individual, household, community and services (Fig. 2). These spheres may in the future the development of interventions for the elderly.

## Results

Participant characteristics have been described in Table 1. The six focus group comprised 7–10 members each, with generally comparable numbers of men and women. The age of the participants ranged from 40 to 80 years; two groups comprised relatively older members (oldest participant was 80 years old) and one group had disproportionately higher number of women. In the health system interviews, participants were generally male (only two providers were female) with an age range of 25–52 years. Interviews were conducted with a range of public and private providers such as government doctor, registered medical practitioners, auxiliary nurse midwife (ANM), pharmacist and private doctor. In the administrator interviews, only one interview could be conducted with a female. Administrators interviewed were young (between 30 and 40 years of age) and at various levels of governance.

We present findings as per the determinants of ageing and morbidity in the conceptual framework.

### Key health ailments

All stakeholders agreed that chronic conditions (hypertension and diabetes), mental distress (loneliness and anxiety) and bone health (including pain) were the major health issues affecting the elderly. Community members perceived hearing and vision disabilities and physical impairments as important concerns for the elderly. Health providers and administrators differed in their view; health providers considered hearing disability as a greater challenge while administrators considered vision disability to be more important. Respiratory health and undernutrition were reported as issues by health providers, but the community and administrators did not consider them to be important concerns.

### Health services

Stakeholders reported a number of health service barriers (e.g. access to diagnostics) faced by the elderly in the villages. All stakeholders agreed that compared to government health services, private providers were available when needed, provided diagnostic support and, in general, better treatment. Health providers and administrators reported that, at present, diagnostic services were limited in the villages. Access to diagnostic services was also a challenge for the elderly due to their frailty, limited mobility and dependence on family members for transport.

*“There is a bus facility; it comes only till the middle of the village but not till here... We have nothing... Facilitator: But these days, there is a bike or a cycle in every house hold… Participant: yes, it is there. But they will take their family members only. If any of their family members suffer with stomach ache, then they will take them to hospital but if other or I get that pain will they take me?”* (FGD, low urbanicity village)

In some cases, health service needs were addressed by mobile health providers (104 mobile health service^3^). Health providers reported that these mobile health vans periodically visited the community and their services were particularly beneficial for the elderly who could not travel long distances. However, community members perceived them as unreliable. Administrators agreed that there was an urgent need to improve health services in the community as the elderly were forced to travel far for simple tests.

*“Establishment of hospital facility is necessary here. Also there should be diagnostic centers as people find it hard to travel a long way for a simple test.”* (Mandal Parishad Territorial Constituency (MPTC) Member, high urbanicity village)

Health providers reported that the elderly expected sensitivity rather than extensive medication from health professionals. However, government health providers did not demonstrate this and were usually perceived as overburdened. Lack of systems for accountability and monitoring of health services in the community were also reasons for patient dissatisfaction.

*“The government hospital does not take much care for the patients. They attend to them and give two medicines, the same medicines are given to everybody, they don’t even hold their hand or check BP for the patients. What the elderly expect is to talk to them and ask how they are. … And it is enough for them even if we don’t prescribe any medicines… It will be good if the government hospital staff also behaves well, but they don’t and cannot do. … Nobody keeps an eye on those doctors. Doctors just come, attend the patients, sit and go. Nobody questions them, why they are not giving medicines, what reason did the patient come to them and what medicines were given.”* (Private Registered Medical Practitioners, low urbanicity village)

### Economic Dimensions

All stakeholders agreed that poverty was a major barrier in accessing nutritious food and medicines. While the community and health providers suggested that there was a need to increase pension amounts, administrators reported challenges with delivery of existing social security schemes (e.g. incorrectly printed names, delays in receiving allotments) that led to delays in receiving entitlements on time. As a result, the elderly were often financially dependent on families or in debt. Community reported that the elderly were often unaware of their entitlements, however, health providers did not consider awareness as a challenge.

*“Her name was printed wrong. Her name is X, they printed it as Y. Few people are facing problems. This person here is also not getting pension. He received his pension for many months, but since two months he is not getting his pension. They started these machines. If they detect all our ten fingers then they give pensions. They will combine these two months pensions and they will give it in next month.”* (FGD, moderate urbanicity village)

### Household factors

Community reported neglect and abuse as key household issues influencing health care use, caregiving and general wellbeing among the elderly. Limited household resources and changing cultural values were considered as reasons for neglect of the elderly within households. Community members shared that the elderly played an important role on cultural occasions, but otherwise felt neglected in household and community matters.

*“During marriages, people give importance to the elderly because they are experienced and have much knowledge about rituals. But otherwise they are not given importance or considered at least.”* (FGD, low urbanicity village)

Community members reported that many elderly had been abandoned by families and relied on their own savings. As a result, loneliness and anxiety were commonly reported mental health issues among the elderly.

*“We stay separately. No children are ready to feed us. We cook for ourselves. With my father in laws’ earnings, we bought a shutter like home. We gave it for rent. With that little amount we adjust ourselves. Our children want that little amount of money also, recently one of son beat my husband for that rent that we get. What can we do madam?”* (FGD, high urbanicity village)

Despite changing values and family dynamics, the community considered caregiving as a family’s responsibility. Economic support for the elderly was to be provided by family members and caregiving during illnesses was seen to be the responsibility of women. Health providers stated that changing norms in the community imposed restrictions on how much villagers or health providers could interfere in family matters.

*“The situations in the villages are in such a way that if we neighbors go and help the elderly people their family members will question us “why are you helping them? Aren’t we there?” Because they think they will be blamed if any neighbors help their elder family members. … one of my friend’s grandfather is a beggar at the temple if at all I go to help him then my friend will question me “aren’t we there why are you doing for him?” And they will scold that old man also. So I feel why should that old man be abused because of me and so I drop myself off from the thought of helping him.”* (Private Registered Medical Practitioners, moderate urbanicity village)

### Influence of Urbanization

Stakeholders perceived urbanization as a positive development for the villages as it was leading to improvements in infrastructure, particularly roads, streetlights, drainage and water supply. Health providers reported that urbanization was leading to increasing socioeconomic status from businesses (e.g. poultry farms), new constructions in the villages and ownership of assets like dish TVs and motor vehicles. However, the benefits of infrastructure improvements such as roads and motor vehicle use were limited for the elderly populations who were frail, dependent on family members and afraid of accidents.

*“There are no accidents in the village but people are scared of the main roads.”* (FGD, low urbanicity village)

We found that the sociocultural environment in the community and within households was undergoing a silent change. Administrators reported that crime was not a major concern in the villages, but instances did occur where the elderly lived alone. Health providers reported that urbanization was changing values in the community leading to frequent instances of familial discord between the elderly and younger family members. Intergenerational disagreements and changing obligations towards older members often led to neglect of health issues of the elderly. Instances of intergenerational discord were often presented as a ‘generation gap’ or difference of opinion. But largely it was felt that these intergenerational differences were induced and magnified by the advent of urbanization in the community. Elderly reported that they felt neglected; community members expressed that the elderly were often angry at their receding authority within households. This often led to feelings of frustration and even despair. We felt that these changes reflected changing sociocultural norms attributed to urbanization, but were also influenced by shifts in power dynamics within households as individuals age and lose authority and say on key household matters.

*“Because of development, there is generation gap between the elders and their grandchildren. ..They get angry very quickly if their children don’t get anything that the elders want. The generation has changed so they are unable to adjust with their daughter in laws. But if you ask me something about their health issues I can tell you but how can I tell about these things?”* (PHC Doctor, moderate urbanicity village)

#### Urbanizing lifestyles and perceived need for health interventions

Changing social norms and urbanization in the community manifested in lifestyle changes in the community. Community reported that increasing uptake of smoking and alcohol use among younger members in the villages was predominantly due to peer influences and was considered disrespectful to elders.

*“if my son comes from there with a cigarette in hand, it will definitely trouble me. Now here we have all elders, if an elder person smoke that doesn’t matter but when a boy smokes it will be like there is no respect to the elders…Smoking starts all because of friends. They see one another and they start. Asks the other also to smoke otherwise he’ll tell him that he will not talk to him. So like that it starts. Even in cool drinks they are mixing alcohol and they are consuming and that too without any differences between boys and girls.”* (FGD, low urbanicity village)

Community reported addiction and relapse as important health issues. These influenced household resources, both directly and through frequent healthcare seeking. Health providers reported that alcohol addiction led to fights within households and alcohol use was seen both among younger and older members in the household.

*“Till he gets cured from his disease he will follow whatever the doctor says but later again he will start following his own ways. Like doctor asks them not to smoke for one or two months till he gets cured from cough problems. After he is cured he again starts smoking.”* (FGD, low urbanicity village)

Health providers and administrators suggested the need for interventions to address health challenges faced by the elderly. These included interventions geared towards management of chronic diseases like type 2 diabetes in the community. Suggestions for interventions included lifestyle modification and increasing awareness of chronic disease management and availability of health facility entitlementss.

*“Another thing is to educate diabetic patients to take nutritional supplementation. It is not followed here in the village. They think they should eat chapatti for breakfast and meals for lunch that’s it. They don’t have any knowledge and they fear eating fruits fearing that it has high sugar content. That way they don’t eat any kind of nutritional foods. Even If we ask them to eat or not to eat few particular things they don’t follow because they prepare it at home and these people eat whatever is available…they should be made aware of the Arogyasree and also educated to take what kind of nutritional supplementation .And also make them learn few exercises for the people suffering from knee and joint pains. They come to us to take medicines for even a very small pain; they have become habituated to this.”* (Private health provider, moderate urbanicity village)

*“Counseling / meetings should be conducted especially for them so that they are made aware of their health, healthy lifestyle and ways of living happily in their old age.”* (Local Councilor, high urbanicity village)

#### Interventions for greater community engagement

Interventions for community engagement were a frequent theme in the focus group discussions and interviews. Administrators reported that they had contributed village land for constructing a hospital which may enhance health service access for the elderly in the future. Communities however perceived that Panchayats were not bothered about the needs of the elderly in the villages and in some instances, the elderly themselves had to demand their entitlements.

*“But they will not look into the problems faced by the elderly sir. The Gram panchayat members are not bothered about the elderly. The elderly, they themselves should go to the Sarpanch (village headman) and say “I am 60 years now, so please grant me pension policy” and then only they will do. Unless and until the older people go and ask, they will not look into their problems”.* (FGD, low urbanicity village)

Administrators suggested that the elderly needed to be involved in political and sociocultural matters in the villages. Suggestions included film shows and cultural programs for the elderly to help in alleviating feelings of loneliness. To increase community engagement, self-help groups could play an important role; so far, their role had been limited to financial schemes.

*“Care and concern for the elderly must be improved in the community. Community gathering for the older people where they can come, sit and talk to each other and spend some time should be arranged such that it helps them to stay happy and healthy.” (Zila Parishad Territorial Constituency (ZPTC) member, high urbanicity village)*

*“Yes, some entertainment like movies, chat with people, should be arranged for the elders. SHG already exists in our village and people get loans from them. As a community, we should talk and mingle with them to them forget and come out of their difficult situations.” (Mandal Parishad Territorial Constituency (MPTC) member, high urbanicity village)*

## Discussion

This study examined perceptions of health needs of the elderly across local stakeholders in an urbanizing community. In doing so, it assessed the influence of urbanization on health and wellbeing of the elderly. Findings from the study not only reflect current challenges, but also indicate issues that will arise with India’s demographic and socioeconomic transition. We summarize three salient findings from the study.

*Firstly*, we noted that challenges related to access of health services in this transitioning rural area were most acutely felt by the elderly. Bodily frailty, limited public transport services and physical and financial dependence on families were principle barriers in accessing medical services such as diagnostics. Existing systems for health care had limited outreach and the public health system was perceived to be overburdened. The role of innovative mobile health services like 104 health van can be crucial for addressing the health services gap, especially related to physical accessibility constraints. Research on rural ageing from developed countries has shown poorer health among older persons in rural compared to urban areas [28], which may be explained by the health services gap. Stakeholders indicated the importance of a number of health conditions including diabetes, bone and mental health, physical impairments, respiratory health and nutritional issues for the elderly. However, these are not included within the current framework of geriatric care programs like the National Program for the Healthcare of the elderly. Stakeholders indicated the need to deliver lifestyle counseling for the elderly to increase awareness and adherence related to risk factors and disease management of diabetes and mental health conditions.

*Secondly*, we found that the influence of urbanization on health of the elderly may be multidimensional and needs greater investigation. Differential urbanization across the rural communities was the force that shaped changing physical infrastructure and social relations. Perceptions of the stakeholders reflected **two dimensions**. At the village level, rural communities welcomed the benefits of urbanization as these improved village facilities but probing showed that the benefits of increasing urbanization were not shared by the elderly with fear of road accidents, crime and social isolation. We found a strong but declining role of social networks (e.g. changing norms in the community leading to neighbors or community members hesitating in what appeared to be family matters) which is likely to have implications for social support and caregiving for the elderly. Greater investigation is needed to understand these influence of urbanization on social networks in transitioning settings and how they impact health and health-seeking for the elderly. Research in rural ageing often highlights medicalized dimensions of ageing, and has overlookedfvillae the influence of social and environmental factors [29, 30]. Secondly, we found the influence of urbanization on within-household dynamics, particularly on the structure and function of families. The decline of multiple-generation households (joint families) to nuclear families and changing social norms within families will lead to an increasing number of older persons will live alone. This has been an area for research among demographers and medical sociologists, but is yet to be mainstreamed as an area of investigation in public health. So far, the health policies assume that the primary provider of support and care, to the elderly is the immediate family. However, with urbanization and migration, as families move away, interventions and services will have to delivered by other providers of care and support. In rural communities, this role may be played by neighbors and community members, religious councils, village councils and even health extension workers. These groups can not only deliver a specific health or social service, but can only provide overarching support to elderly living alone, those in conflict with their households and others in need of social engagement. This requires further examination of the financial, healthcare and social services that are needed to address health needs and care-giving gaps for the elderly.

*Finally*, all stakeholders suggested strengthening the role of community institutions in delivery and monitoring of programs. Community members proposed that local governance institutions (village councils or *Panchayats*) needed to play a more effective role in relation to health services and social security schemes for the elderly. The role of village councils in health monitoring, service delivery and programme planning for reproductive and child health services has been one of the pillars of the national health programme [31–33]. Community based institutions including self help groups have also played a role in women’s empowerment and community asset creation [34, 35]. Kerala, a southern Indian state that is rapidly ageing and ahead of other Indian states in the epidemiological transition [36], is increasingly providing new models for care related to palliative care and chronic disease management [37]. There is a need to study these models and their impact on health and well being of older persons as these provide valuable lessons to other Indian states and LMICs in general. In this study, in the absence of a local database for schemes disbursed, health providers suggested that *Panchayats* could conduct periodic surveys on health needs and vulnerabilities of the elderly. This would help in monitoring delivery of pensions and other schemes in the villages. As schemes are increasingly linked through a unique identification number in India [38], the role of community based institutions in overseeing the needs of the elderly and ensuring delivery of entitlements and services could be enhanced.

The National Program for Healthcare of the elderly (NPHE) was designed to create an ‘architecture’ for ‘healthy and active ageing’ [17]. The program mandates a range of services for geriatric care to be delivered at sub-center, primary, community and district level hospitals [17]. The early roll-out phase of the program presents an opportunity to understand and amalgamate stakeholder perspectives in order to deliver health services that the elderly need. The present study indicates a diversity of issues brought forth by local stakeholders that are not being addressed presently in health services. In particular, specific components of the health policy related to geriatric care and ageing in India need to be strengthened. This study is only a first step in highlighting issues faced by ageing populations from the perspective of different stakeholders in an urbanizing rural setting. It brings to light some of the many challenges faced and the changing landscape of issues from the view of those who will be implementing, delivering and receiving services. Instead of a top-down policy maker perspective, we tried to present the view from the community, which can highlight the challenges that will be faced as new programs and policies are implemented to influence the wellbeing of the elderly in the future. The question of how we perceive the needs of older populations needs to be examined across other contexts in India and LMICs that are witnessing ageing. In particular, the question has resonance in both rural and urban settings and in different geographies with unique challenges that will influence the development of ageing-friendly policies and services.

Bronfenbrenner’s Ecological Systems theory provided the landscape for analyzing and categorizing perspectives and interventions suggested by different stakeholders (Fig. 2). The constructs in the theory of micro-, meso-, exo- and macrosystems were translated in the study as household, community and services, with the larger upstream determinant of urbanization. These spheres developed a priori, were confirmed in the analysis of data and were linked through themes and codes from the analysis (Fig. 1). We referred to them as Social Determinants of Ageing in a bid to understand the broader structural factors influencing ageing and development leading to morbidity (physiological, impairments, mental and cognitive health) and dependence on others (physical, financial, caregiving and emotional). These determinants are not only adding to our understanding of ageing in rural and urbanizing contexts, they arealso providing insight into the direction of services and interventions – and the levels at which these services and interventions may be planned and delivered. Interventions around families and household pertained mostly to care and financial support; at the community level, stakeholders recommended community-level governance surveys, financial support through pensions and community avenues for institutional support. At the health services level, stakeholders recommended greater access to preventive and diagnostic services, increasing access to medicines and interventions around adherence, and an overall view to improve the quality of health services. The upstream determinant of urbanization was considered a force that influenced both family and community. Based on stakeholder view, we recognized that knowledge of the pathways and impact on chronic disease and health seeking among the elderly was lacking. At the same time, there was a greater need to identify infrastructure services that would improve access to health care on the elderly as well as understand the changing influences and services needed to intervene on dimensions of social networks, social support and community norms. These spheres were seen to interact and influence one another, on their way to impacting the older person in the community.

We acknowledge two *limitations* of the study. Firstly, we could not conduct separate focus group discussions for the elderly, family and other villagers due to feasibility and cultural reasons. Additionally, we did not stratify the views of elderly and other members in the focus group discussions. We were not interviewing the elderly and felt that the view of the community captured a composite view of issues and perspective of the community. It is possible that if probed separately, differences of opinion or contradictions may emerge. However, the purpose of the study was to view the community view of ageing to understand expectations, interests and issues faced. Secondly, this was an exploratory study to bring forward diverse dimensions related to health needs of elderly. We were interested in a composite view of how the different stakeholders perceived ageing and needs of the elderly, and not merely in medicalizing or focusing on specific health ailments in the community. Several of these dimensions require detailed investigation which was not possible during this study.

## Conclusion

This study recommends urgent action to strengthen the health services infrastructure in India, with particular focus on outreach and lifestyle counseling for the elderly to promote healthy ageing. Understanding the multidimensional influences of urbanization for health and wellbeing of the elderly through further research will also provide effective strategies for social policy interventions for the elderly.

## Additional file

Additional file 1: Table S1. Differences in characteristics of urbanicity across villages in the study. (DOCX 61 kb)

## Abbreviations

APCAPS: Andhra Pradesh Children and Parents Study
FGD: Focus group discussion
MPTC: Mandal Parishad Territorial Constituency
NPHCE: National Program for Healthcare of Elderly
PHC: Primary Health Center
ZPTC: Zila Parishad Territorial Constituency

## Footnotes

1 Families in south Asia have typically comprised cohabiting multiple generations (‘joint’ families) with within household hierarchies and role relations. With socioeconomic transitions, changes in occupational structures, social norms, urbanization and migration, families are transitioning to ‘nuclear’ households comprising two adults and children. The joint family has sometimes been associated with Hindu religion (hence the phrase ‘Hindu joint family’); however, this phenomenon has applied to south Asia and often transcends religious boundaries.

2 Gram Sabha is an elected local body created at the village level that has jurisdiction on basic governance and social matters of the village

3 104 is a mobile health van that visits the community once every fortnight and can provide basic preventive care services and OPD. This is an initiative of the State government and is being conducted by a non-profit organization based on a Public Private Partnership model. This van makes fortnightly or monthly visits to villages and provides free medication. A doctor who travels in the van does a BP measurement and basic diagnostics as well.
