# Supplementary material for: Health needs, access to healthcare, and perceptions of ageing in an urbanizing community in India: a qualitative study
Source: BMC Geriatr. 2017 Jul 19;17:156. doi: 10.1186/s12877-017-0544-y (PMC5517927; doi:10.1186/s12877-017-0544-y)
Supplement: Additional file 1: Table S1. — Differences in characteristics of urbanicity across villages in the study. (DOCX 61 kb) [file 12877_2017_544_MOESM1_ESM.docx]

**Additional file 1: Table S1**

| Urbanicity of village (as per night time light intensity) | Number of villages in the study | Population (as per a 2013 survey) | Distance (km) from Hyderabad city |
| --- | --- | --- | --- |
| Low | 4 | 1931-3112 | 41-54 |
| Medium | 2 | 1466-6630 | 40-44 |
| High | 6 | 1262-11361 | 29-56 |
